# Psychological and Emotional Impact of Patients Living in Psychiatric Treatment Communities during Covid-19 Lockdown in Italy

**DOI:** 10.3390/jcm9113787

**Published:** 2020-11-23

**Authors:** Jessica Burrai, Paolo Roma, Benedetta Barchielli, Silvia Biondi, Pierluigi Cordellieri, Angelo Fraschetti, Alessia Pizzimenti, Cristina Mazza, Stefano Ferracuti, Anna Maria Giannini

**Affiliations:** 1 Department of Dynamic and Clinical Psychology, Sapienza University of Rome, Via Degli Apuli 1, 00185 Rome, Italy; benedetta.barchielli@gmail.com; 2 Department of Psychology, Sapienza University of Rome, Via dei Marsi 78, 00185 Rome, Italy; pierluigi.cordellieri@uniroma1.it (P.C.); angelo.fraschetti@uniroma1.it (A.F.); annamaria.giannini@uniroma1.it (A.M.G.); 3 Department of Human Neuroscience, Sapienza University of Rome, Viale dell’Università 30, 00185 Rome, Italy; paolo.roma@uniroma1.it (P.R.); silviabiondi14@gmail.com (S.B.); stefano.ferracuti@uniroma1.it (S.F.); 4 SRTR Villa Costanza, gruppo Sage, Via Belmonte 74, 00079 Rome, Italy; info@alessiapizzimenti.it; 5 Department of Neuroscience, Imaging and Clinical Sciences, G. d’Annunzio University of Chieti-Pescara, Via dei Vestini 33, 66100 Chieti-Pescara, Italy; cristina.mazza@uniroma1.it

**Keywords:** Covid-19, psychiatric patients, stress, anxiety, depression, coping, worry, risk perception, mental illness

## Abstract

Most studies on well-being during the COVID-19 pandemic have focused on the mental health of the general population; far less attention has been given to more specific populations, such as patients with mental illness. Indeed, it is important to examine the psychiatric population, given its vulnerability. The present study aimed at assessing the psychological and emotional impact of isolation on patients in Residential Rehabilitation Communities, compared to healthy controls. A questionnaire was administered cross-sectionally on an online survey platform and both psychiatric patients and healthy controls accessed via a designed link. The results showed significant differences between psychiatric patients and controls on Anxiety, Stress, Worry, and Risk Perception variables. Psychiatric patients scored lower on Stress compared to healthy controls and higher on Anxiety, Perceived Risk of getting infected with COVID-19 and Worry about the emergency situation. The results showed that, during the Italian lockdown, psychiatric patients living in residential communities received unbroken support from peers and mental health professionals, maintained their usual medication treatment, and were informed of COVID-19 consequences. This finding provides insight into the differences between residential and healthy populations and highlights the importance of continuous support for psychiatric patients, especially during stressful situations such as a pandemic.

## 1. Introduction

Over the past century, there have been several pandemics, with many devastating consequences. In a pandemic situation, when there is no pre-existing immunity to pandemic pathogens, no effective pharmacological treatment, and no vaccine, individuals’ only preventive health measure is to practice good hygiene (e.g., wash hands) and social distancing [1]. Thus, in the wake of the global spread of SARS-CoV-2 and its associated disease (COVID-19), governments have implemented significant containment measures, including quarantines and lockdowns and the closure of schools, offices, and non-essential shops [2]. 

Research on individual and community responses to restrictive measures in the context of previous infectious disease outbreaks (e.g., Severity Acute Respiratory Syndrome [SARS]; H1N1; Ebola; Middle East Respiratory Syndrome [MERS]; equine influenza) have studied the psychological effects of isolation, social distancing, and lack of physical contact. The findings have demonstrated a significant psychological impact on individual well-being, characterized by higher levels of Anxiety, Depression, and Stress [3,4,5,6,7,8]. Recent studies conducted during the COVID-19 pandemic have shown similar consequences of the lockdown on psychophysical wellness in the general population [9,10,11,12,13,14,15]. 

Most studies on well-being during the COVID-19 pandemic have focused on the mental health of the general population; far less attention has been given to more specific populations, such as patients with mental illness [16]. In this context, it is important for researchers to examine the psychiatric population, given its vulnerability. It has already been shown that both older adults and those who are immunocompromised (including psychiatric patients) are at greater risk for developing complications when infected with COVID-19 (e.g., comorbidity with other medical disorders, see, e.g., [17]). 

In addition, the co-occurrence of mental illness and substance use disorders [18] as risk factor for COVID-19 infection, and risk adverse outcomes related to cardiovascular, pulmonary and metabolic disease associated to an extent with chronic use of alcohol and other drugs need to be taken into account [19].

Moreover, social isolation is part of the symptomatology of many psychiatric disorders. For this reason, at first it could be assumed that lockdown policies could reduce the stress related to compliance with social norms; on the other hand, it should be considered that the long-term outcomes could be an increase of rumination and decompensation due to a possible exacerbation of loneliness and despair relating to social isolation [20].

Furthermore, the psychiatric population may increase their suffering in reaction to forced isolation and compromise their ability to adequately understand the emergency and implement appropriate containment behaviors. 

Very few studies have addressed the effects of comprehensive restrictive public health measures on psychiatric patients. To the best of our knowledge, during the SARS epidemic, only one study [21] investigated the effect of the epidemic on the clinical state of psychiatric inpatients with schizophrenia, finding them to present lower levels of Anxiety, Depression, and Fear compared to a control group of health staff. The authors explained these findings as indicative of the in-patients’ greater denial of the importance and personal relevance of the epidemic. 

With respect to the COVID-19 pandemic, contrasting results have emerged, showing a higher sensitivity in psychiatric patients due to pandemic-related Stress [22]. Hao et al. [22] studied the psychological impact of the pandemic on psychiatric patients and healthy controls during the peak of viral spread in China. The psychiatric patients (who had been diagnosed with Major Depressive Disorder, Generalized Anxiety Disorder, Panic Disorder, and/or mixed Anxiety and Depressive Disorder) showed significantly higher levels of Post-Traumatic Stress Disorder (PTSD), Depression, Anxiety, Stress, and insomnia, compared to the healthy controls. More specifically, 31.6% of the psychiatric sample fulfilled the diagnostic criteria for PTSD, while 23.6% showed moderate to severe anxiety symptoms and 22.4% showed moderate to severe depressive symptoms. Finally, more than 25.0% of the psychiatric patients suffered from moderately severe to severe insomnia [22]. The authors suggested that the worse outcomes shown by their psychiatric patient group may have been caused by their lack of access to mental health services during the emergency.

During the COVID-19 pandemic, people with mental illness were also shown to demonstrate stronger emotional responses compared to the general population, as a result of their higher susceptibility to Stress [16]. Psychiatric patients were also found to experience higher levels of Distress, which could play a mediating role in elevating levels of Anxiety [23]. 

All of the abovementioned studies were conducted online, using samples of in-patients at hospitals and psychiatric units. To the best of knowledge, no prior study has assessed psychiatric patients in a rehabilitation community. In such communities, psychiatric patients have adjusted to conditions of less personal autonomy and community engagement; they no longer attend school and/or internships and they do not leave the facility to visit family and friends. Thus, the typical residential conditions are similar to a quarantine or lockdown situation.

The present study aimed at assessing the psychological and emotional impact of isolation on patients in these psychiatric communities, compared to healthy controls. In more detail, we wondered whether there might be significant differences between psychiatric patients and healthy controls during the COVID-19 lockdown in relation to Coping Style, Risk Perception, and Worry, as well as levels of Depression, Anxiety, and Stress. Furthermore, we sought to identify potential risk and protective factors for psychological distress, taking into account sociodemographic variables (i.e., age, gender, education), Worry, Risk Perception, and—particularly—the presence or absence of a psychiatric diagnosis.

## 2. Experimental Section

### 2.1. Participants

A questionnaire was administered cross-sectionally on an online survey platform, which participants (both psychiatric patients and healthy controls) accessed via a designed link. The respondents were 82 Italian psychiatric patients, living in two rehabilitation communities in the Lazio region during the COVID-19 lockdown, and 106 healthy control subjects, recruited online and randomly chosen from a larger sample on the basis of mean age. The two communities are accredited by the National Health Service and provide healthcare assistance through qualified personnel 24 h per day. Various professional figures work closely with psychiatric patients within the community: psychiatrists, educators, psychologists, nurses and social assistants. During the lockdown, all the professionals continued to work in the community guaranteeing psychiatric patients’ continuity of care and treatment. In both communities, an attempt was made to maintain a link with the patients’ affections and families through remote communication systems, strengthening the Internet connection and helping psychiatric patients with video calls. Several meetings have been held within the community between the mentioned professionals in order to organize specific therapy groups for the psychiatric patients. Positive reinforcement techniques were used to encourage participation in therapy groups to prepare the patients to face social isolation and emotional flattening.

The psychiatric patients were aged 18 years or older and had been diagnosed with at least one psychotic disorder; healthy controls were aged 18 years or older and had no psychiatric diagnosis. All participants voluntarily responded to the anonymous survey and indicated their informed consent. The procedures were clearly explained, and participants could interrupt or quit the survey at any point without providing explanation. As regards the questionnaire administered to psychiatric patients, the first items were intended for the community administrators who helped patients respond to the measure. The remaining items were administered for patient self-report.

Five psychiatric patients and six healthy controls were excluded from the analysis because they did not complete the entire survey. The final sample consisted of 177 participants. The psychiatric patient sample comprised 77 participants: 51 males (*M_years_* = 47.29; *SD* = 13.26) and 26 females (*M_years_* = 45.27; *SD* = 12.03), aged 22–73 years, with a mean age of 46.61 (*SD* = 12.81). Most psychiatric patients did not have children and were either retired or recipients of state support. The healthy control sample comprised 100 participants: 50 males (*M_years_* = 48.38; *SD* = 12.79) and 50 females (*M_years_* = 44.42; *SD* = 9.81), aged 20–69 years, with a mean age of 46.40 (*SD* = 11.52). No significant difference was found between groups in age [*F* (1,176) = 0.013, *p* = 0.909], while significant differences were found in gender [*χ^2^* (1) = 4.679, *p* = 0.031] and education [*F* (1,176) = 14.796, *p* ≤ 0.001] (see Table 1).

Data were collected between April and May 2020, before the end of the lockdown period in Italy. Expedited ethics approval was obtained from the Institutional Board of the Department of Human Neuroscience, Faculty of Medicine and Dentistry, “Sapienza” University of Rome (IRB-2020-6), in conformity with the principles of the Declaration of Helsinki.

Descriptive statistics are reported in Table 1.

Most psychiatric patients (93.5%) received training and education on COVID-19 and its transmission by the referred community, hence we assessed patients’ knowledge about the COVID-19 pandemic using three items (i.e., “How did you become aware of the spread of COVID-19?”; “What is COVID-19?”; “Did you participate in community training sessions on this health emergency?”). Each of these items enabled more than one response option to be indicated. Furthermore, the first two items were also administered by the healthy control group, in order to assess where they had retrieved information about the virus and their awareness about it. Possible responses to the first item differed between groups, since the psychiatric patients were living in the rehabilitation communities and had contact with the operators. Table 2 reports the response frequency of these items.

### 2.2. Materials 

The Depression, Anxiety and Stress Scale–21 items (DASS-21) [24] was used to assess participants’ mental health. The DASS-21 is a set of three self-report scales designed to measure the emotional states of Depression, Anxiety, and Stress. All subscales are rated on a 4-point Likert scale ranging from 0 (never) to 3 (almost always). In our sample, Cronbach’s alphas were 0.82, 0.83, and 0.87 for the Depression, Anxiety, and Stress Subscales, respectively.

The Brief Resilient Coping Scale (BRCS) [25] is a four-item questionnaire designed to capture highly adaptive Stress coping tendencies. It is rated on a 5-point Likert scale ranging from 1 (does not describe me at all) to 5 (describes me very well). Total scores range from 4–20, with scores of 4–13 indicating low resilient coping, scores of 14–16 indicating medium resilient coping, and scores of 17–20 indicating high resilient coping. In the present sample, the BRCS showed a Cronbach’s alpha of 0.64.

Risk Perception was assessed through two variables, perceived severity and perceived likelihood, using items adapted from Cho and Lee [26] and Liao et al. [27]. Perceived severity was measured through four items (e.g., “If I got COVID-19, it would be severe”) and perceived likelihood was measured through two items (“How likely is it that you will get COVID-19 in this period?”). Twelve questions were assessed on a 5-point Likert scale ranging from 1 (not likely at all) to 5 (certain). In the present sample, the items showed good reliability, with Cronbach’s alpha of 0.72.

Worry was assessed using six items (e.g., “In the past week, you have been worried about contracting the COVID-19”) designed to measure Worry about the present emergency situation, risk of contracting COVID-19, and perspectives on the future. Items were assessed on a 5-point Likert scale ranging from 1 (hardly) to 5 (extremely). The items showed good reliability, with Cronbach’s alpha of 0.63.

### 2.3. Statistical Analysis 

Descriptive statistics of both the psychiatric patient and the healthy control groups were collected in order to summarize the variables. Analysis of variance (ANOVA) tests were run to identify differences between groups in their scores for Risk Perception, the BRCS, Worry, and DASS-21 Depression, Anxiety, and Stress. Two multiple linear regressions were performed using the enter and stepwise methods, respectively, to compute associations between sociodemographic variables (i.e., age, gender, education), the presence/absence of diagnosis, Risk Perception, and Worry (used as independent variables); and DASS-21 Anxiety and Stress (used as dependent variables). DASS-21 Depression was excluded because no differences were found between groups. All statistical analyses were conducted using the software package SPSS, version 25.

## 3. Results

### 3.1. Between Groups Comparison (ANOVAs)

As reported in Table 3, psychiatric patients demonstrated lower scores on DASS-21 Anxiety, BRCS, Risk Perception, and Worry. In more detail, ANOVAs showed a significant difference between groups on DASS-21 Anxiety and Stress, Risk Perception, and Worry. In particular, psychiatric patients scored lower on Stress and higher on Anxiety, Perceived Risk of getting infected with COVID-19 and Worry about the emergency situation, compared to healthy controls.

No significant differences were found between groups on DASS-21 Depression and the BRCS.

### 3.2. Multiple Linear Regression

The final multiple linear regression model accounted for a significant portion of the variance in DASS-21 Anxiety [R2 = 0.27 (Adj R2 = 0.25), F-change = 8.997, *p* = 0.003]. Lower age, together with a psychiatric diagnosis and higher scores on the Worry measure, were found to be significant predictors of DASS-21 Anxiety. Risk Perception (*B* = 0.052, *p* = 0.764) was excluded (see Table 4).

The final multiple linear regression model accounted for a significant portion of the variance in DASS-21 Stress [*R*^2^ = 0.28 (*Adj R*^2^ = 0.26), *F-*change = 30.920, *p* ≤ 0.001]. Lower age, a psychiatric diagnosis, and higher scores on the Worry measure were found to be significant predictors of DASS-21 Depression. Risk Perception (*B* = 0.118, *p* = 0.492) was excluded (see Table 5).

Since no differences were found between groups on the DASS-21 Depression subscale, it was excluded, and the regression analysis was not carried out for this specific variable.

## 4. Discussion

The present study evaluated the impact of the COVID-19 pandemic on Risk Perception, Worry, Depression, Anxiety, Stress, and Coping Strategies among Italian psychiatric patients living in a rehabilitation community, compared to healthy controls. The results showed significant differences between psychiatric patients and controls on Anxiety, Stress, Worry, and Risk Perception variables. Concerning the DASS-21, psychiatric patients scored lower on all three subscales than the comparative clinical sample studied by Bottesi et al. [24], who registered mean scores of 5.5 (*SD* = 4.6), 7.7 (*SD* = 5.6), and 8.9 (*SD* = 4.2) for the Depression, Anxiety, and Stress subscales, respectively. Although psychiatric patients in the current study generated lower DASS-21 scores, all scores were within a normal range, according to Lovibond and Lovibond’s [28] version of the scale. In contrast, other studies of both non-residential psychiatric patients and the general population have identified higher DASS-21 scores during the COVID-19 lockdown [11,22]. 

The lower scores registered in the present study could be explained by the residential care condition of our study sample. As reported by Tansella [29], it is possible to hypothesize that residents of psychiatric rehabilitation communities, unlike psychiatric inpatients in a hospital setting, experience greater support from mental health workers and peers, present higher perceived security, and, above all, enjoy minimal coercion and maximal freedom. Moreover, unlike the population of non-residential psychiatric patients who were forced to reduce their access to psychiatric care and pharmacological and psychological support during the lockdown [30], the residential psychiatric patients in our sample were able to maintain their typical levels of care and support. This could explain their DASS-21 scores in the normal range. 

Residence in a psychiatric rehabilitation community during lockdown may have been an important factor in limiting psychiatric patients’ increase in depressive, anxious, and stressful symptoms. However, psychiatric patients did still show higher levels of Anxiety relative to healthy controls. While this significant difference might be explained by the impact of COVID-19 on psychopathology and mental health [31], it could also be related to psychiatric pathology. In fact, there is a higher prevalence of Anxiety disorders in people diagnosed with schizophrenia or other spectrum psychotic disorders, with 6.3% of this population presenting at least one lifetime Anxiety disorder [32,33,34].

In the present study, psychiatric patients presented with lower Stress compared to controls. This result could relate to their pre-existing adaptation to conditions of restricted personal freedom (i.e., limits imposed by the psychiatric community), in contrast to healthy controls, who were not accustomed to the limitations of freedom required by the lockdown. Considering that the lockdown implied separation from loved ones, loss of freedom, and boredom, higher Stress levels would be expected, as supported by research on the impact of quarantine on the general population [35].

In addition, psychiatric patients obtained a medium score on the BRCS, demonstrating a medium level of resilience [25]. Resilience and gratitude are considered protective mechanisms in conditions of trauma [36], and the resilience exhibited by the present psychiatry patients may have made them less vulnerable to COVID-19 stressors. 

Furthermore, psychiatric patients scored higher than healthy controls on the Perceived Risk of getting infected with COVID-19. They received training and education on COVID-19 and its transmission by their rehabilitation community, and this might explain their higher perception of risk relative to healthy controls, as measured by the relevant survey items (e.g., “If I got COVID-19, it would be severe”).

Moreover, psychiatric patients showed higher levels of Worry than the control group, particularly with respect to worries about their health and contraction of the virus [37]. Overall, the presence of a psychiatric diagnosis and higher scores on the Worry measure were found to be significant predictors of DASS-21 Anxiety and Stress. As testified by the cognitive behavioral model [38], Worry may produce negative interpretations of information. For example, Internet search results (e.g., graphic images of the surge of contagion, alarmism, fake news, conspiracy theories) could exacerbate fear, Stress, and Anxiety and, in some people, manifest nightmares and intrusive thoughts concerning COVID-19 [39]. 

It is worth noting that the present sample mainly consisted of younger adults (psychiatric patients: *M* = 46.61, *SD* = 12.81; healthy controls: *M* = 46.40, *SD* = 11.52). Previous studies on the COVID-19 epidemic have found a different age and gender association with Perceived Risk and Worry, with older adults demonstrating a higher risk perception and younger adults demonstrating greater Worry [40]. In the present study, both psychiatric patients and healthy controls scored higher on Worry than Risk Perception. Our results also found an association between having a psychiatric disorder and increased Anxiety and Stress.

To the best of our knowledge, the present study was the first to provide data on a population of psychiatric patients who, during the lockdown in Italy, lived within a psychiatric community that provided continuous support and care. However, the study has some limitations. First, we were limited in our ability to draw comparisons between psychiatric patients and healthy controls, due to significant differences in gender and educational level. Previous studies have investigated the impact of education level on individual responses to COVID-19, showing that, in healthy controls, a lower level of education is correlated with a lower awareness of COVID-19; this may be due to limited access to health information, reduced access to health care, and increased financial burden [41]. Furthermore, this study was conducted in only two communities in the Lazio region and may not reflect trends observed in similar contexts. The present study also used an observational design; therefore, no assumptions of causation can be made, as baseline evaluations for the psychological variables investigated were not available. Despite these limitations, this is, to the best of our knowledge, the first study to have examined the psychological impact of the threat of COVID-19 on psychiatric patients living in rehabilitation communities during the lockdown.

## 5. Conclusions

While quarantine and lockdown measures have been successful in limiting the spread of COVID-19, they have also resulted in isolation and loneliness, which might trigger or aggravate pre-existing mental illnesses [42]. However, despite their pervasive and long-lasting psychological impact, such measures still remain the best available containment strategy, and may be considered the lesser of two evils.

The present findings paint a general picture of the psychological impact of COVID-19 on the psychiatric residential population, in comparison with the general population, and provide a baseline for future research on the impact of COVID-19 on individuals and specific populations.

The results showed that, during the lockdown in Italy, psychiatric patients living in residential communities received unbroken support from peers and mental health professionals, maintained their usual medication treatment, and were informed of the possible consequences of COVID-19 and which strategies to implement to protect against contagion. Consequently, they had significantly lower levels of Stress than the non-residential psychiatric population. This finding provides insight into the differences between residential and non-residential psychiatric populations and highlights the importance of continuous support for psychiatric patients, especially during stressful situations such as a pandemic.

## Figures and Tables

**Table 1:** Descriptive statistics of the study sample.

		Psychiatric Patients	Healthy Controls
Characteristic	Group	*N* (%) = 77	*N* (%) = 100
Age	*M* (*SD*)	46.61 (12.81)	46.40 (11.52)
	Min–Max	22–73	20–69
Gender	Female	26 (33.8%)	50 (50%)
	Male	51 (66.2%)	50 (50%)
Education	Primary school diploma	6 (7.8%)	4 (4%)
	Middle school diploma	37 (48.1%)	28 (28%)
	High school diploma	28 (36.4%)	47 (47%)
	Graduate	5 (6.5%)	21 (21%)
	Unknown	1 (1.3%)	0 (0%)

**Table 2:** Descriptive statistics of the sample, in regard to knowledge about COVID-19.

Questions	Options	Psychiatric Patients *N (%)*	Healthy Controls *N (%)*
Information about COVID-19 retrieved from	Media	60	82
	Social networks	13	37
	Family	9	18
	Community operators *	37	-
	Community hosts *	7	-
	Organized communication from community staff *	25	-
	Other	4	2
What is COVID-19	Seasonal flu	2	5
	Respiratory syndrome	39	42
	Respiratory insufficiency	35	38
	High temperature	18	15
	Fake news	0	2
	Terrorist attack	2	0
	Other	9	3
Participation in formative meetings about COVID-19 *	Yes	72 (93.5%)	-
	No	5 (6.5%)	-

Note. * indicates responses/questions administered only to the psychiatric patients.

**Table 3:** Between groups comparison (ANOVAs).

	Psychiatric Patients N = 77	Healthy Controls N = 100	F	p	parη2
DASS-21 Depression	4.58 (4.10)	5.77 (5.53)	2.488	0.116	0.014
DASS-21 Anxiety	**4.04 (4.39)**	**2.66 (3.98)**	**4.776**	**0.030**	**0.027**
DASS-21 Stress	**5.94 (4.83)**	**7.92 (5.53)**	**6.252**	**0.013**	**0.034**
BRCS	14.58 (3.03)	15.13 (2.84)	1.513	0.220	0.009
Risk Perception	**41.96 (7.77)**	**18.50 (2.87)**	**772.276**	**<0.001**	**0.817**
Worry	**15.80 (3.79)**	**8.88 (2.05)**	**207.898**	**<0.001**	**0.543**

Note. BRCS: Brief Resilient Coping Scale. Emboldened results were found to be significant, with *p* < 0.05.

**Table 4:** Multiple linear regression of DASS-21 Anxiety.

	Unstandardized Coefficients *B*	Std. Error	Standardized Coefficient *B*	*t*	*p*
**(Constant)**	−1.559	2.407		−0.648	0.518
Age	**−0.047**	**0.023**	**−0.133**	**−1.985**	**0.049**
Gender	−0.993	0.579	−0.117	−1.716	0.088
Education	−0.281	0.362	−0.055	−0.776	0.439
Diagnosis, ref. no	**2.549**	**0.850**	**0.300**	**2.999**	**0.003**
Worry	**0.609**	**0.098**	**0.627**	**6.215**	**<0.001**

Note. Emboldened results were found to be significant, with *p* < 0.05.

**Table 5:** Multiple linear regression of DASS-21 Stress.

	Unstandardized Coefficients *B*	Std. Error	Standardized Coefficient *B*	*t*	*p*
**(Constant)**	2.520	3.009		0.837	0.404
Age	**−0.098**	**0.029**	**−0.222**	**−3.345**	**0.001**
Gender	−1.252	0.723	−0.117	−1.731	0.085
Education	−0.490	0.453	−0.076	−1.083	0.280
Diagnosis, ref. no	**6.413**	**1.062**	**0.599**	**6.037**	**<0.001**
Worry	**0.681**	**0.123**	**0.556**	**5.561**	**<0.001**

Note. Emboldened results were found to be significant, with *p* < 0.05.
